# Effects of square dance exercise on cognitive function in elderly individuals with mild cognitive impairment: the mediating role of balance ability and executive function

**DOI:** 10.1186/s12877-024-04714-x

**Published:** 2024-02-15

**Authors:** Heng Wang, Zhengguo Pei, Yangyang Liu

**Affiliations:** College of Physical Education, Henan Normal University, 453007 Xinxiang, China

**Keywords:** Square dance exercise, Mild cognitive impairment (MCI), Balance ability, Executive function, Chain mediating effect

## Abstract

**Background:**

Square dancing is a kind of aerobic fitness exercise without environmental restrictions that yields many benefits for physical and mental health; this exercise is popular among middle-aged and elderly people in China and in these populations in other countries. This study aimed to evaluate the effects of square dance exercise on the overall cognitive function of elderly individuals with mild cognitive impairment (MCI) and to research its mechanisms.

**Methods:**

A total of 60 elderly people with MCI (60–69 years old) without square dance experience were selected and randomly divided into an experimental group (*n* = 30) and a control group (*n* = 30). The experimental group participated in square dance exercise for 12 weeks, while the control group maintained their original lifestyle habits. One week before and after the intervention period, the overall cognitive function, physical fitness, and executive function of both groups were measured.

**Results:**

According to the results, square dance exercise directly improved the overall cognitive function of elderly individuals with MCI and indirectly affected overall cognitive function through the mediating effects of balance ability and executive function.

**Conclusions:**

Square dance exercise represents a nonpharmacological intervention for the prevention and treatment of MCI. Importantly, it is best to combine this exercise with other forms of physical exercise and comprehensive treatment programs such as cognitive training, social interaction, and psychological intervention to realize its maximum effect.

## Introduction

Mild cognitive impairment (MCI) is a state of cognitive impairment between cognitive function with normal aging and dementia [1, 2]. Its core symptom is the decline of cognitive function, including impairment or reduction in memory, attention, executive function, language logic, and visuospatial skills [2, 3]. MCI is a heterogeneous syndrome. Based on the number of impaired cognitive domains, MCI can be divided into single-domain MCI and multiple-domain MCI [4]. Single-domain MCI refers to only one cognitive domain being mildly impaired while multiple-domain MCI refers to mild impairments in multiple cognitive domains, but it is not severe enough to be diagnosed as dementia. In addition, according to different core clinical characteristics, MCI can also be classified into subtypes such as amnestic, executive, language/semantic, attentional, and visuospatial/perceptual [5]. The diagnosis of MCI requires standardized cognitive testing to rule out cognitive decline caused by other factors. The global prevalence of MCI is between 9.6% and 21.6%. Since approximately 10–15% of people with MCI develop Alzheimer’s disease each year, the condition of MCI is considered best stage for preventive intervention [6–9].

### Effects of physical exercise on cognitive function

Physical activity or exercise has long been associated with improvements in cognitive function [10]. In addition, exercise is an important lifestyle factor for maintaining the health of elderly individuals [11] and plays a crucial role in improving the health and quality of life of these individuals as well as enabling engagement in social activities [12, 13]. At present, an increasing number of studies have shown that the cognitive function of elderly individuals can be improved by training or even reversed to some extent [14, 15]. Researchers refer to this improvement as neural plasticity and cognitive plasticity. Previous studies have focused on the direct influence of physical exercise on cognitive function (i.e., the direct effect of physical exercise). However, to some extent, they have overlooked the possible factors mediating the relationship between physical exercise and cognitive function and thereby ignored potential indirect effects. In addition, previous studies have focused on physiological changes, and few studies have focused on the mechanism of cognitive changes. To confirm that physiological benefits and cognitive benefits are equally important results of physical exercise for elderly individuals, this study examined the direct and possible indirect effects of physical exercise on cognitive function in elderly individuals with MCI in terms of physical fitness and cognitive changes.

### The mediation role of physical fitness and executive function

For elderly individuals with MCI, physical exercise interventions mainly include aerobic exercise with increased oxygen supply, resistance exercise that increases muscle mass and strength, and combined exercises (i.e., multimodal training, including aerobic, strength, balance, and dual-task training). From the perspective of physical performance, these exercises improve one or more dimensions of individual physical fitness. Cognitive performance is mainly based on the neurotrophic hypothesis, which postulates that during exercise, individuals release several neurotrophic molecules that stimulate hippocampal neurogenesis, cerebrovascular generation and monoamine synthesis, leading to lasting neurological changes in the brain that enhance brain integrity and function and improve cognitive function. For example, aerobic exercise increases brain volume in the temporal lobe and prefrontal cortex of the brain, including gray matter and white matter [16]. It can lead to a 2% increase in the volume of the hippocampus, a brain region crucial for learning and memory. Resistance training is associated with increased levels of insulin growth factor-1 (IGF-1) in the blood [17, 18], and moderate intensity resistance training can reduce the progression of white matter lesions in the brain.

This shows that changes in physical fitness and cognition are interrelated. Changes in one aspect are bound to lead to changes in the other. Whether positive or negative, these changes ultimately lead to changes in cognitive function. Therefore, it can be inferred that changing physical fitness through physical exercise can improve cognitive function to some extent. Some studies have shown that physical health is significantly negatively correlated with the performance of certain cognitive tasks (such as shifting tasks) [19]. For example, there is a negative correlation between weight status and academic performance, and agility is positively correlated with cognitive flexibility and inhibitory control. Similar results have been reported in studies among children and adolescents with normal weight [20, 21]. Moreover, there was a positive correlation between health and executive function performance. Therefore, it can be inferred that physical fitness mediates the relationship between physical exercise and cognitive function.

As one of the higher-order cognitive functions, executive function (EF) is responsible for controlling and coordinating various specific cognitive processes [22, 23]. Executive function, which includes inhibitory, updating and shifting, has consistently been a hot topic in the fields of neuropsychology, cognitive psychology and cognitive neuroscience [21]. Executive function is mainly supported by the frontal lobe (especially the prefrontal lobe). Compared with other brain regions, the frontal lobe exhibits a more rapid decline with aging [24, 25] in terms of cortical atrophy, white matter degradation, reduced functional connectivity and changes in functional activation patterns [26–28]. The executive decline hypothesis of cognitive aging proposes that the decline or impairment of executive function with aging (alterations in the frontal lobe) is the main cause of the decline or impairment of people’s daily cognitive function (memory, reasoning, and visuospatial, emotional regulation skills) [29–31]. Several studies [32–35] have found that different forms and durations of physical exercise, such as aerobic, resistance, long-term exercise, or acute exercise, can improve executive function and cognitive function in older adults with MCI. Executive function is a higher-order cognitive function; thus, improvements in executive function improve overall cognitive function [36]. A study by Qin demonstrated that inhibitory control and working memory predicted the cognitive ability of high school students [37]. Moreover, a meta-analysis of 91 studies by Verhaeghen and Salthouse [38] reported that processing speed and working memory play important mediating roles in cognitive function and the aging process, and the same findings were described by Li Deming et al. [39]. After applying a stratified regression analysis to data from 1,350 adults, the effects of processing speed and working memory on cognitive function during aging were found to explain approximately 87% and 76% of the variance, respectively, and the combined effects of the two explained approximately 94% of the variance. It can be inferred that executive function mediates the relationship between physical fitness and cognitive function.

Physical exercise, which improves physical fitness and executive function, may have an important role in brain maturation and the optimal developmental trajectory of cognitive function. According to Information Processing Theory, in the early stage of physical exercise, the brain’s processing of information directly depends on the characteristics of stimuli or sensory information from external inputs (i.e., bottom-up processing). This processing not only has a positive impact on physical fitness but also on the development of executive function. Moreover, there may be a link between the physical fitness and executive function. For example, some studies have found that upper and lower extremity strength and balance positively correlated with executive function in older adults [40–42], and that cardiovascular health of older adults are significantly correlated with their attention, executive function and overall cognition [43]. In particular, older adults with lower levels of cardiorespiratory fitness have been found to exhibit poorer executive function [44], and older adults’ muscle strength has a domain-specific effect on their working memory [45]. Therefore, there may be a chain mediating effect of physical exercise on cognitive function that follows the following path: physical exercise → physical fitness → executive function → cognitive function.

### Characteristics and effects of square dance exercise

**Fig. 1:**
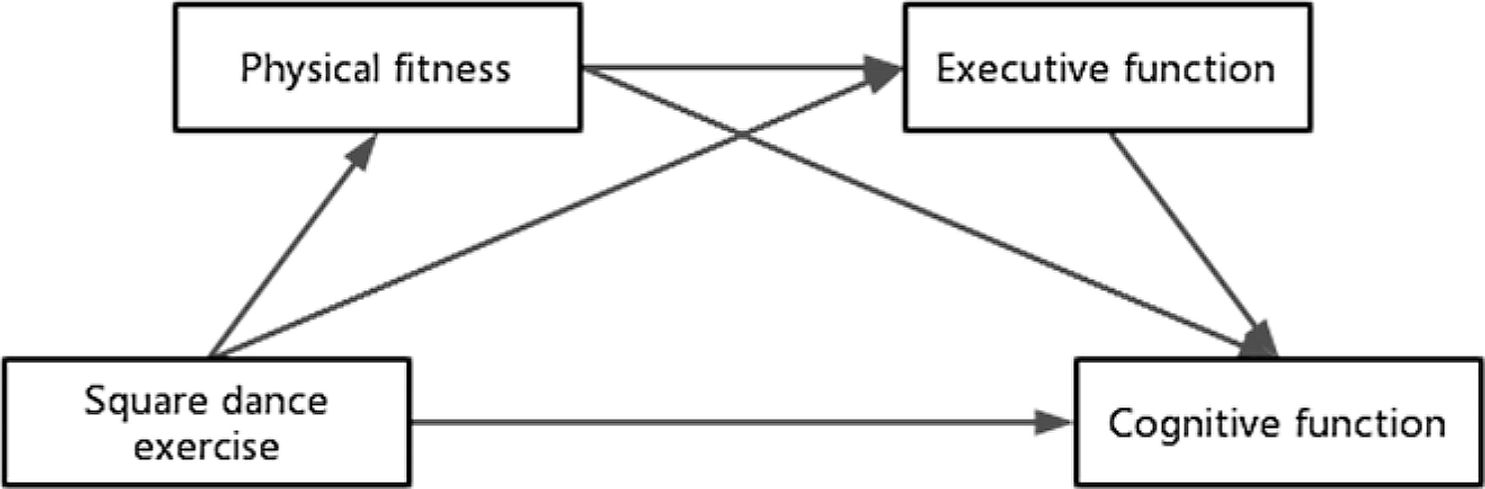
Hypothetical path model of this study

Some studies have reported that ballroom dancing [46] and traditional Greek dancing [47] have positive impacts on the overall cognition, mood, balance and quality of life of elderly people with MCI. However, in the Chinese population with MCI, ballroom dance and traditional Greek dances are not widely known, and it is difficult to encourage widespread use due to their complexity and cultural unfamiliarity. The widespread popularity of Chinese square dancing has introduced a novel exercise option for elderly individuals in China. It represents an aerobic fitness activity presented through song and dance, characterized by an inclusive, self-entertaining, extensive, and mass-appealing dance style. This style seamlessly incorporates elements from traditional Chinese culture [48]. This form of dance consists of music, a group of dancers, and one or more leaders. It is usually performed in large public places in a variety of forms, such as gymnastics, folk dance, disco, and modern dance. It is characterized by its simplicity, freedom from environmental restrictions and strong social functions [49, 50]. Moreover, studies have shown that square dance exercise can promote social interactions with peers and alleviate loneliness; importantly, these benefits are also known to improve health during aging [51, 52]. Therefore, we selected a Chinese square dance intervention and analyzed the relationships among square dance exercise, physical fitness, executive function and overall cognitive function; and also explored the influence of square dance exercise on the overall cognitive function of elderly individuals with MCI and its underlying mechanism. We proposed the following hypotheses. Hypothesis 1: Square dance exercise improves the overall cognitive function of elderly individuals with MCI. Hypothesis 2: Physical fitness mediates the relationship between square dance exercise and overall cognitive function. Hypothesis 3: Executive function mediates the relationship between square dance exercise and overall cognitive function. Hypothesis 4: Square dance exercise has a chain mediating effect on cognitive function along the following path: square dance exercise → physical fitness → executive function → overall cognitive function. The hypothetical model is shown in Fig. 1.

## Experimental methods

### Selection and grouping of experimental subjects

Subjects were selected through explanation, posters and leaflets at a large nursing home in Xinxiang, Henan Province, China. The team led by Professor Hongxing Zhang from Xinxiang Medical College conducted clinical diagnosis and cognitive function assessment to screen MCI elders. The specific content and process are as follows: Firstly, a detailed clinical medical history is collected, and the elders and their family members are asked about their observation of cognitive decline. The symptoms and duration of suspected cognitive decline are identified, while factors such as head injury history, cerebrovascular disease history, drug or alcohol abuse history that can cause secondary cognitive impairment are excluded. Then, neurological examinations (visual and auditory examinations, motor system examinations, sensory system examinations, balance and cerebellar function examinations, etc.) will be performed on these elders to rule out the impact of other neurological diseases on cognitive function. Finally, elders with no clear pathological changes were asked to complete the Montreal Cognitive Assessment (MoCA) scale, and those with a total score below 26 were classified as elders with MCI.

Meanwhile, elders with the following conditions were also excluded: (1) chronic disease that could affect exercise ability, (2) recent treatment or medication for cognitive impairment or depression, (3) other diseases that could affect cognitive function, (4) visual or hearing impairment that affected communication, and (5) regular exercise in the past six months (30 min/d for at least 3 d/w). The sample size was calculated using G Power software [53–55]. With effect size (Cohen’s f = 0.25), 80% power, and 1% Class I error, we estimated that a minimum of 48 participants would be required. Therefore, 60 subjects aged 60–69 years were selected after completing the Montreal Cognitive Assessment (MoCA) and clinical diagnosis. After obtaining consent from elderly individuals and their guardians, the subjects were randomly divided into the experimental group (*n* = 30) and the control group (*n* = 30) using the SPSS 27.0 “visual binning” method.

The experimental group engaged in square dance exercise 40 min/session, 4 times/week for 12 weeks. The square dance is the 17th set fitness routine of the “Happy Dance of the Chinese Dream Team” This is a moderate-intensity marching aerobics routine composed of twelve segments, encompassing movements for the upper limbs, shoulders, chest, waist and abdomen, and lower limbs. Each segment consists of 1–3 simple movements. Upper limb movements primarily include straight-arm circles and swinging the arms straight in various directions. Shoulder movements predominantly involve internal and external shoulder rotation, as well as upward and downward movements. Chest movements require participants to swing both arms backward to stretch the chest. Waist and abdominal movements mainly include slight lateral bends, waist twists, and hip turns. Lower limb movements mainly include leg lifts, knee raises, and kicks in various directions. The dance lasts for 30 min, with each segment accompanied by a musical piece. Participants stand in a formation of 5 columns and 6 rows, led by a researcher serving as the dance leader. The dance activities take place in the outdoor square, with indoor sessions in the activity hall during adverse weather conditions. Each exercise session lasts for 40 min, comprising a 5-minute warm-up (finger exercises, joint movements, and stretching), 30 min of dance, and 5-minute cool-down (deep breathing and stretching). In order to ensure the adequate implementation of experimental intervention, during the experiment, we arranged for one person to lead the dance and another person to observe the participants’ practice, including their attendance, following the rhythm of the music, and the quality of completing the movements. After the end of the session, interviews were conducted to evaluate the participants’ mastery of square dance to confirm their serious cooperation with the practice.

**Fig. 2:**
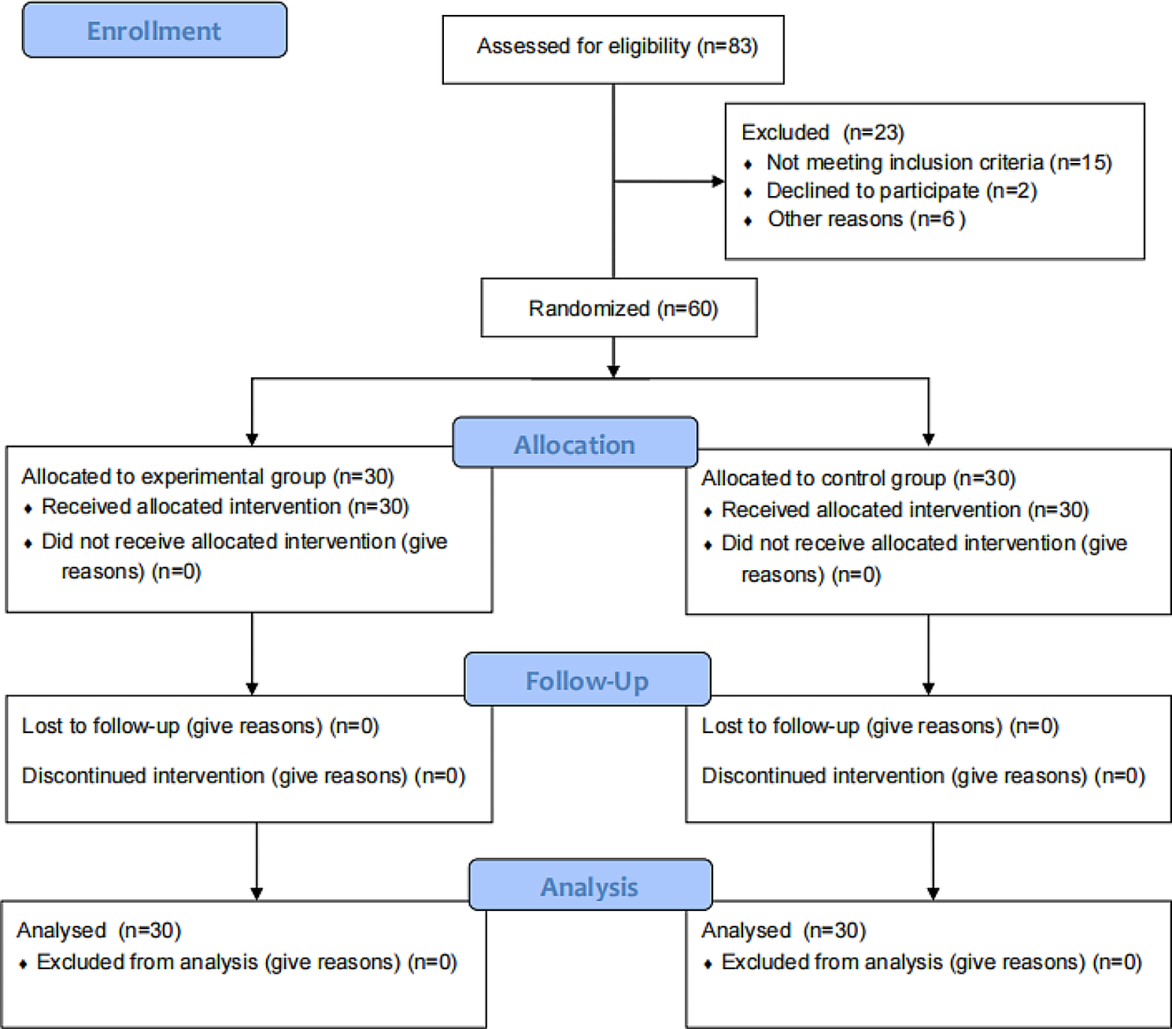
Flow diagram of screening, intervention, measurement, and final sample included in data analysis

The control group did not engage in additional physical exercise and maintained their original lifestyle habits. Physical fitness, executive function and overall cognitive function were evaluated one week before and after the intervention period for the both groups (Fig. 2). This study was approved by the Academic Committee of Henan Normal University. The elderly individuals with MCI who participated in this study received a gift worth 100 RMB after the experiment.

### Overall cognitive function test

The MoCA is commonly used to evaluate cognitive function. In this study, it was used to screen subjects in terms of overall cognitive function. The total score on the MoCA ranges from 0 to 30, and scores ≥ 26 are considered normal. Eight aspects of cognition are assessed: visuospatial skills, naming, memory, attention, language, abstraction, delayed recall and orientation. The higher the score is, the better the overall cognitive function. The MoCA has a sensitivity of 0.93, specificity of 0.87, test-retest reliability of 0.92, and Cronbach’s α of 0.86 [56].

### Measurements of physical fitness

Physical fitness was measured with China’s National Physical Fitness Standard Manual (Seniors) [57]. This evaluation includes measurements of strength (hand grip strength; kg); speed (15-m shuttle run; s), flexibility (sit-and-reach test; cm), and balance (standing on one foot with the eyes closed; s).

### Measurements of executive function

Performance on three domains of executive function was tested on a laptop computer, and the tests were programmed with E-Prime 2.0 software. All subjects completed the 3 tests, and each test included a corresponding practice session. When the practice accuracy was ≥ 80%, they entered the formal test (average accuracies of the 3 tests: 83.1%, 84.5% and 81.8%). The specific tests used to evaluate the three domains are as follows.

Inhibitory function [58]: The Stroop task was used. This test consists of a color judgment task and a word judgment task. In the formal experiment, the instructions were presented, and the subjects were asked to perform the word meaning judgment or color judgment. The subjects pressed any key to formally start the experiment. In the test task, a fixation cross (“+”) appeared in the center of the screen for 500 ms, and then Chinese characters (“red” or “green”) appeared for 1,500 ms. This task involved two conditions: congruent and incongruent. In the congruent condition, the color and meaning of the two Chinese characters were the same (for example, the “green” character was presented in green, and the “red” character was presented in red). In the incongruent condition, the Chinese characters had different colors and meanings (e.g., the “green” character was presented in red, and the “red” character was presented in green). When the Chinese characters appeared, two types of judgment were performed. When the “green” character was shown in green and the “red” character was shown in red, subjects pressed the “F” key; when the “green” character was displayed in red and the “red” character was displayed in green, subjects pressed the “J” key. The congruent and incongruent trials appeared in a random order, with 60 trials each. The interval between trials was 1,000 ms. The test result was the average reaction time in the incongruent condition minus the average reaction time in the congruent condition. The smaller the difference in reaction times was, the better the inhibitory ability. In addition, accuracy and speed were needed.

Updating function [59]: The n-back test was used, which included two tasks. In the 1-back test, a number between 1 and 9 appeared on the computer screen, and each number was presented separately in the center of the computer screen. The presentation duration of the stimulus number was 800 ms, and the interval between two numbers in a trial was 3,000 ms. The subjects were instructed to look at these numbers carefully for 60 trials. When the second number appeared, subjects compared this number with the previous number. If the numbers were the same, subjects pressed “F”; if they were different, subjects pressed “J”. In the 2-back test, a number between 1 and 9 appeared on the screen, and each number was presented separately in the center of the computer screen. The presentation duration of the stimulus number was 1,000 ms, and the interval between numbers in a trial was 3,000 ms. The subjects were instructed to look at these numbers carefully for 60 trials. When the third number in a trial appeared, subjects compared it to the first number in a trial. If the numbers were the same, they pressed “F”; if they were different, they pressed “J”. The test score was the average reaction time. The shorter the reaction time (in ms) was, the better the updating ability. Accuracy and speed were needed.

Shifting function [59, 60]: The more-odd shifting test was used. In this test, a series of numbers were presented one by one in the center of the computer screen, with a presentation time of 3,000 ms and an interval between numbers of 3,000 ms. The subjects were asked to make judgments regarding the numbers (between 1 and 9, excluding 5). There were three tasks. Task A involved a large/small judgment: red numbers were presented, and if the number was less than 5, subjects pressed the “F” key; if the number was more than 5, they pressed the “L” key. Task B involved an odd/even judgment: black numbers were presented; if the number was odd, subjects pressed “F”, and if the number was even, subjects pressed “L”. Task C was a combination of the above two tasks and involved a large/small and odd/even judgment. In other words, subjects made a large/small judgment if the numbers presented were red and made an odd/even judgment if the numbers presented were black. Tasks A and B involved 16 trials each, and the number of trials in Task C was converted to 32 trials, for a total of 64 trials. The final test score was the average reaction time of the shifting task minus the average reaction time of the corresponding nonshifting task. The smaller the difference in reaction time was, the better the shifting ability. Accuracy and speed were needed.

### Statistical analysis

1. SPSS 27.0 was used to carry out repeated-measures analysis of variance (ANOVA) for the 2 (pre-test and post-test) × 2 (experimental group and control group) design.
2. The model fitting was set and tested in SPSS Amos 28.0. software. The SPSS macro program Process plug-in compiled by Hayes was used to test the mediating effect. The bootstrap method was used to test whether there was a mediating effect for physical fitness and executive function between square dance exercise and overall cognitive function. The number of samples was set to 2000, and the default confidence interval was 95%.
3. In order to avoid false positives and a high error rate caused by a large number of statistical tests, multiple tests were performed using the Bonferroni correction, and the multiple tests were corrected to a significant level of *p* = 0.0125, with a p value of 0.0125 being the original p value of 0.05/4.
4. K-fold cross-validation: the dataset was partitioned into a homogeneous 6 subsets, and 5 of these subsets were used to construct model and the other 1 subset for validation with repeating the process 6 times to ensure that each subset acts as a test set once and calculating the average of the model performance metrics over the 6 iterations to prevent model overfitting.

### Minimal clinically important difference (MCID)

We used distribution-based and anchor-based approaches to estimate the MCID of square dance exercise. Distribution-based MCID is calculated as one-half of the SD of all participants at pre-intervention [61–62]. For the anchor-based approach, we selected MoCA as the external criterion. The anchor-based MCID is calculated as the average score change of participants whose score change is equal to or greater than one-half of the SD of MoCA at pre-intervention [63].

### Homogeneity test of participants before the experiment

To determine whether the overall cognitive level, physical fitness and executive function of all subjects were similar before the experiment, an independent-sample t test was used (Table 1). The results revealed no significant differences in physical fitness, cognitive function or executive function between the experimental group and the control group (*p* > 0.05).

**Table 1:** Descriptive statistics (M, SD) of the two groups of subjects before the intervention

Variable	Dimension	Experimental group	Control group	t	*p*
OCF	MoCA	19.40 (1.73)	19.37 (1.59)	0.08	0.086
PF	Strength (kg)	24.93 (5.71)	27.28 (4.12)	-1.83	0.404
	Flexibility (cm)	0.80 (5.49)	2.03 (4.97)	0.12	0.466
	Balance (s)	4.47 (2.60)	4.27 (2.48)	0.31	0.761
	Speed (s)	8.10 (0.43)	8.06 (0.54)	0.34	0.733
EF	Inhibitory (ms)	197.40 (58.67)	226.03 (56.51)	-1.93	0.059
	Updating (ms)	839.03 (73.65)	850.93 (61.85)	-0.68	0.501
	Shifting (ms)	328.10 (76.70)	313.07 (54.62)	0.87	0.386

Note: OCF = overall cognitive function, PF = physical fitness, EF = executive function

## Results

### The intervention effects of square dance exercise

To verify the effects of square dance exercise on the overall cognition, executive function and physical fitness of elderly individuals with MCI, repeated-measures ANOVA was adopted. The descriptive statistics of each variable are shown in Tables 2 and 3.

The ANOVA results showed that overall cognitive function (F_(1, 58)_ = 55.013, *p* < 0.00025, ${{\eta }}_{\text{P}}^{2}=0.487$); inhibitory (F_(1, 58)_=94.630, *p* < 0.00025, ${{\eta }}_{\text{P}}^{2}=0.620$), updating (F_(1, 58)_=38.739, *p*<0.00025, ${{\eta }}_{\text{P}}^{2}=0.400$), and shifting domains of executive function (F_(1, 58)_=10.327, *p* < 0.0025, ${{\eta }}_{\text{P}}^{2}=0.151$); and balance ability of physical fitness (F_(1, 58)_=78.615, *p* < 0.00025, ${{\eta }}_{\text{P}}^{2}=0.575$) displayed a significant effect of the time by group interaction in elderly individuals with MCI. Further simple effects analysis of the above variables revealed significant differences between groups after the intervention in overall cognitive function, inhibitory, updating, shifting and balance ability; specifically, these abilities were better in the experimental group than in the control group. The strength (F_(1, 58)_=2.55, *p*>0.0125, ${{\eta }}_{\text{P}}^{2}=0.042$), flexibility (F_(1, 58)_=0.136, *p*>0.0125, ${{\eta }}_{\text{P}}^{2}=0.019$) and speed (F_(1, 58)_=0.010, *p*>0.0125, ${{\eta }}_{\text{P}}^{2}=0.000$) domains of physical fitness did not display a significant effect of the interaction between time and group. In conclusion, square dance exercise improved the overall cognitive level; inhibitory, updating and shifting of executive function; and balance ability of physical fitness in elderly individuals with MCI.

**Table 2:** Descriptive statistics (M, SD) and ANOVA results regarding overall cognitive function and executive function in the two groups before and after the intervention period

Group	Time	MoCA score	Executive function		
			Inhibitory (ms)	Updating (ms)	Shifting (ms)
Experimental group	Pre-test	19.40 (1.73)	197.40 (58.67)	839.03 (73.65)	328.10 (76.70)
	Post-test	23.33 (1.09)	133.53 (68.03)	744.05 (99.98)	305.40 (78.05)
Control group	Pre-test	19.37 (1.59)	226.03 (56.51)	850.93 (61.85)	313.07 (54.62)
	Post-test	19.33 (1.77)	225.43 (63.14)	869.33 (99.56)	317.63 (55.42)
Group by time interaction: F_(1, 58)_	Pre vs. post comparison	55.013***	120.060***	38.739***	10.327**
${{\eta }}_{\text{P}}^{2}$		0.487	0.674	0.400	0.151

* *p* < 0.0125, ** *p* < 0.0025, *** *p* < 0.00025 (The Bonferroni correction was used to conduct multiple tests and obtained the significance level *p* = 0.0125 after multiple test correction, and the p value 0.0125 was the original p value 0.05/4. The same below.)

**Table 3:** Descriptive statistics (M, SD) and ANOVA results regarding physical fitness of the two groups before and after the intervention period

Group	Time	Physical fitness			
		Strength (kg)	Flexibility (cm)	Balance (s)	Speed (s)
Experimental group	Pre-test	24.93 (5.71)	0.80 (5.49)	4.47 (2.60)	8.10 (0.43)
	Post-test	25.35 (5.50)	0.97 (5.43)	10.70 (4.04)	8.12 (0.48)
Control group	Pre-test	27.28 (4.12)	2.03 (4.97)	4.03 (2.48)	8.06 (0.49)
	Post-test	27.31 (4.13)	1.83 (4.94)	4.20 (3.96)	8.07 (0.53)
F_(1,58)_ (Interaction)	Pre vs. post comparison	2.550	0.136	78.620***	0.010
${{\eta }}_{\text{P}}^{2}$		0.042	0.019	0.575	0.000

### Correlations and multiple linear regression results of each variable

First, to verify the relationships among physical fitness, executive function and overall cognitive function after the intervention, post-test data were subtracted from pre-test data to obtain variables with significant differences, and Pearson correlation analysis was used. As shown in Table 4, inhibitory (*p* < 0.001), updating (*p* < 0.001) and shifting (*p* < 0.05) were significantly negatively correlated with overall cognitive function; balance ability (*p* < 0.001) was significantly positively correlated with overall cognitive function.

**Table 4:** Correlations among significant outcome variables

	Cognitive function	Inhibitory	Updating	Shifting	Balance ability
Cognitive function	-				
Inhibitory	-0.918***	-			
Updating	-0.783***	0.684***	-		
Shifting	-0.305*	0.545***	0.502***	-	
Balance ability	0.852***	-0.763***	-0.620***	-0.569***	-

* *p* < 0.05, ** *p* < 0.01, *** *p* < 0.001

Second, to further verify the relationships among the variables, overall cognitive function was included as the dependent variable, and square dance exercise, inhibitory, updating, shifting and balance ability were included as independent variables in a multiple linear regression analysis with the stepwise method. As shown in Table 5, the regression model that included square dance exercise, balance ability and inhibitory as independent variables and overall cognitive function as the dependent variable was significant (F = 198.832, *p* = 0.001, R^2^ = 0.905). Square dance exercise, balance ability and inhibitory significantly affected overall cognitive function. The regression model that included square dance exercise, balance ability and updating as independent variables and overall cognitive function as the dependent variable was significant (F = 165.890, *p* < 0.001, R^2^ = 0.881). Square dance exercise, balance ability and updating significantly affected overall cognitive function. The regression model that included square dance exercise, balance ability and shifting as independent variables and overall cognitive function as the dependent variable was significant (F = 119.774, *p* < 0.001, R^2^ = 0.833). In conclusion, square dance exercise, balance ability and shifting significantly affected overall cognitive function.

**Table 5:** Multiple regression analysis of overall cognitive function with square dance exercise, balance, and executive function

Dependent variable	Model	Unstandardized coefficient		Standardized coefficient	t	*p*	Unstandardized 95%confidence interval		F	R	R^2^
		β	SE	Beta			LL	UL			
OCF	Constant	3.519	0.973		3.263	0.001	1.635	5.451	198.832	0.943	0.905
	SDE	3.532	1.042	0.246	3.391	0.001	1.395	5.698			
	Balance	0.485	0.110	0.283	4.328	0.000	0.245	0.728			
	Inhibitory	-0.089	0.012	-6.721	-6.434	0.000	-0.123	-0.059			
OCF	Constant	6.453	0.872		7.965	0.000	4.779	8.274	165.890	0.932	0.881
	SDE	5.910	0.932	0.443	5.783	0.000	3.184	7.561			
	Balance	0.592	0.122	0.313	4.918	0.000	0.33	0.781			
	Updating	-0.023	0.006	-0.310	-5.095	0.000	-0.053	-0.022			
OCF	Constant	8.027	0.970		8.523	0.000	5.647	9.451	119.774	0.912	0.833
	SDE	7.732	1.12	0.521	6.081	0.000	5.646	9.083			
	Balance	0.586	0.141	0.332	4.132	0.000	0.278	0.618			
	Shifting	-0.032	0.013	-0.131	-2.603	0.012	-0.047	-0.005			

Note: OCF = overall cognitive function, SDE = square dance exercise, LL = low limit of the confidence interval, UL = upper limit of the confidence interval (The multiple regression model was trained using a 6-fold cross validation method)

### Structural equation modeling

A structural equation model was constructed to test the mediating roles of physical fitness and executive function in the relationship between square dance exercise and overall cognitive function. In the model, square dance exercise was included as an independent variable; balance ability, inhibitory, updating and shifting were included as mediating variables; and overall cognitive function was included as the dependent variable. The fit of the overall model was tested with Amos 28.0. As shown in Table 6, all the fit indices of the three models were within the statistical range, indicating that the mediation model fit the data well.

**Table 6:** Indicators of model fit

Treg	Mediator	Absolute fit index		Value-added fitting index			Parsimonious fit index	
SC		χ^2^/df	RMSEA	IFI	TLI	CFI	PGFI	PNFI
JS		< 5	< 0.08	> 0.9	> 0.9	> 0.9	> 0.5	> 0.5
Fitting effect	BI	1.73	0.025	0.994	0.969	0.971	0.534	0.591
	BU	1.77	0.032	0.997	0.981	0.980	0.550	0.630
	BS	1.89	0.035	0.907	0.901	0.981	0.509	0.510

Note: SC = specific classification, JS = judgment standard, BI = balance inhibitory, BU = balance updating, BS = balance shifting

**Fig. 3:**
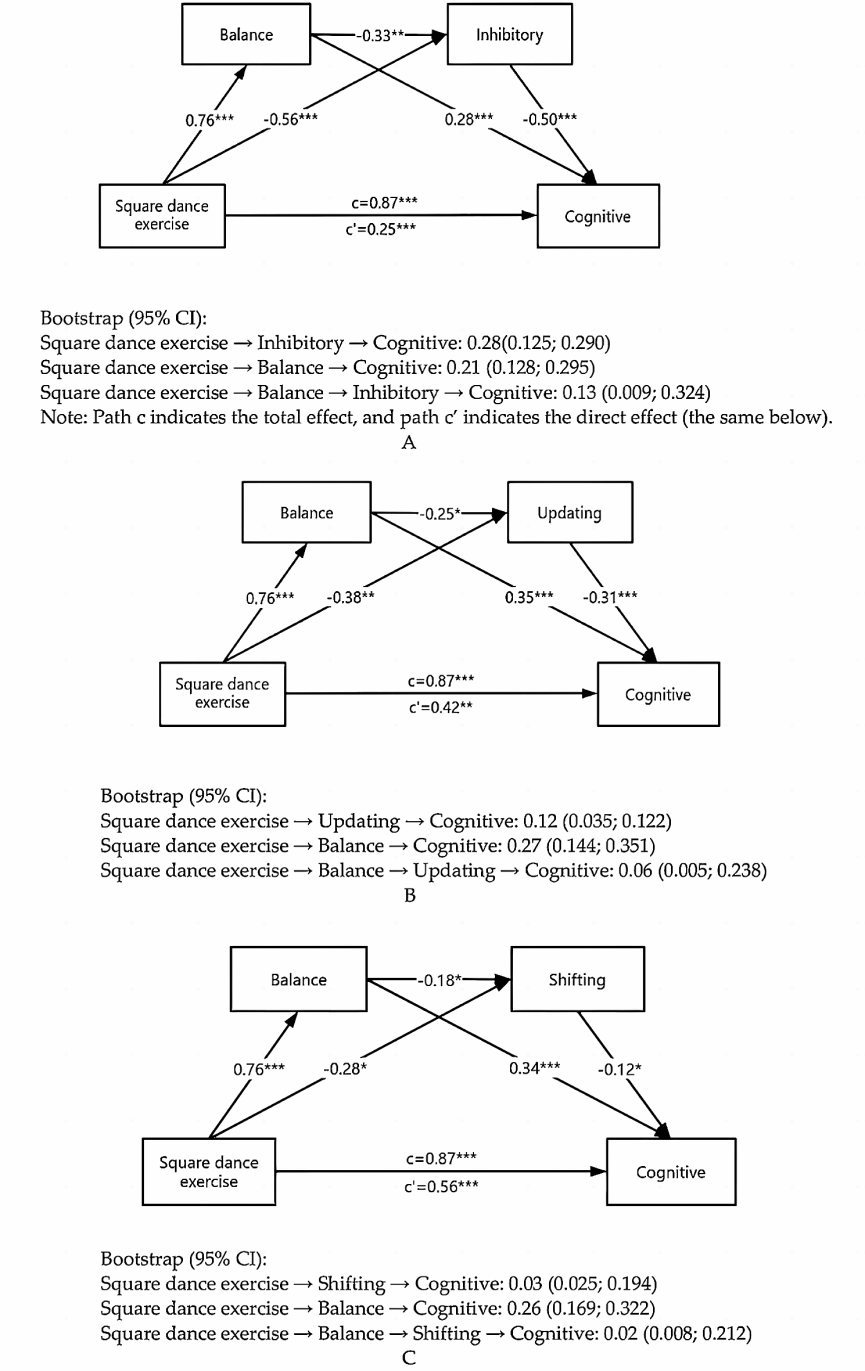
Mediation models of the relationships among square dance exercise, balance ability, types of executive function (inhibitory, updating, or shifting), and overall cognitive function

As shown in Fig. 3A, the total effect of square dance exercise on cognitive function was 0.87 (95% CI= [0.807, 0.932]); this confidence interval excluded 0, indicating the presence of a significant total effect. The direct effect was 0.25 (95% CI= [0.082, 0.461]). The indirect effect of the path from square dance exercise → balance ability → cognitive function was 0.21 (95% CI= [0.128, 0.295]). The indirect effect of the path from square dance exercise →inhibitory→cognitive function was 0.28 (95% CI= [0.125, 0.290]). The indirect effect of the path from square dance exercise → balance ability → inhibitory → cognitive function was 0.13 (95% CI= [0.009, 0.324]). The above results indicate that square dance exercise had a direct effect on balance ability and inhibitory and improved cognitive function; the relationship between square dance exercise and cognitive function was mediated by balance ability and inhibitory.

Figure 3B shows that the total effect of square dance exercise on cognitive function was 0.87 (95% CI= [0.807, 0.932]). The direct effect was 0.42 (95% CI= [0.236, 0.427]). The indirect effect of the path from square dance exercise → balance ability → cognitive function was 0.27 (95% CI= [0.144, 0.351]). The indirect effect of the path from square dance exercise → updating → cognitive function was 0.12 (95% CI= [0.035, 0.122]). The indirect effect of the path from square dance exercise → balance ability → updating → cognitive function was 0.06 (95% CI= [0.005, 0.238]). The above results indicate that square dance exercise had a direct effect on balance ability and updating, which can improve cognitive function. Moreover, these results demonstrated mediating effects of balance ability and updating on the relationship between square dance exercise and cognitive function.

Figure 3C shows that the total effect of square dance exercise on cognitive function was 0.87 (95% CI= [0.807, 0.932]). The direct effect was 0.56 (95% CI= [0.435, 0.731]). The indirect effect of the path from square dance exercise → balance ability → cognitive function was 0.26 (95% CI= [0.169, 0.322]). The indirect effect of the path from square dance exercise → shifting → cognitive function was 0.03 (95% CI= [0.025; 0.194]). The indirect effect of the path from square dance exercise → balance ability → shifting → cognitive function was 0.02 (95% CI= [0.008; 0.212]). These results suggest that square dance exercise had a direct effect on balance ability and shifting, which improved cognitive function. In addition, the results showed that balance ability and shifting mediated the relationship between square dance exercise and cognitive function.

### Minimal clinically important difference (MCID)

The anchor-based MCID was calculated as the average score change of participants whose improved score was equal to or greater than one-half of the SD of MoCA at pre-intervention. One-half of the SD of MoCA were 0.865 for the experimental group (Table 2). Since partial points on the MoCA were not possible, we rounded the value of the criterion to 1 point. For the experimental group, the anchor-point-based MCID was calculated for 13 participants with a change in score greater than or equal to 1. Of the participants in the experimental group, 43% (*n* = 13) had a meaningful improvement in their MoCA score from baseline.

## Discussion

In this study, the effects of square dance exercise on overall cognitive function in elderly individuals with MCI were investigated. The research aims were as follows. First, we aimed to verify the effect of square dance exercise on overall cognitive function in elderly individuals with MCI, and meet the minimal clinically important difference. Second, we aimed to explore the effect of square dance exercise on physical fitness and executive function and, in turn, on the overall cognitive function of elderly individuals with MCI. Finally, we aimed to determine the path from square dance exercise to improvements in overall cognitive function in elderly individuals with MCI by constructing a structural equation model.

### Influence of square dance exercise on the overall cognitive function of elderly individuals with MCI

The findings suggested that square dance exercise improved overall cognitive function in elderly individuals and can be used as a non-pharmacological intervention for the prevention and treatment of MCI. These benefits may be due to the multi-modal nature of square dance exercise, which includes body movement, auditory perception, visual perception and social interaction. These different stimuli can facilitate connections and communication among neurons and enhance neural plasticity, thus improving cognitive function. Research has shown that physical exercise increases the number of neurons and connections of neurons in the brain and improves cognitive functions such as attention, working memory, decision-making and creativity. Therefore, physical exercise can help individuals better understand and apply knowledge, promoting cognitive development and learning.

A study [64] found that 6 weeks of aerobic exercise can increase gray matter volume in the hand motor cortex, striatum, and cerebellum of elderly people. The striatum may be associated with central executive functions (e.g. attention and processing motor-related information) during sports, and the cerebellum is associated with motor learning and refinement in movement during sports. For example, Taubert et al. [65] found that exercise training can change connectivity patterns and activity in cerebellar regions, improving physical coordination through functional MRI scans. Therefore, it can be inferred that physical exercise can promote individual motor ability and motor-related cognitive abilities by changing cerebellar structure.

A systematic evaluation study [66] and an intervention study [67] have found that square dance exercise has a positive impact on the cognitive function of the elderly, and can improve the cognitive function of the elderly in working memory, cognitive flexibility, language ability, visuospatial ability and attention. In addition, square dance exercise can also improve the psychological state and quality of life of the elderly, reduce their anxiety and depression, and enhance their social communication ability and physical health [54, 68]. Therefore, the improvement of the social ability of the elderly may also be another factor causing the improvement of their cognitive function because it was found that the increase of individuals’ social activities has a positive effect on their cognitive function [69].

### The mediating effect of balance ability on the relationship between square dance exercise and overall cognitive function

Balance is the ability of the body to automatically adjust and maintain a posture when moving or being subjected to external forces. It depends on the body’s ability to coordinate and integrate proprioceptive and visual stimuli from the vestibular organs and muscles, tendons and joints. In older people, balance ability gradually declines, which can lead to falls and other unexpected events and affect cognitive performance. Therefore, improving the physical balance of elderly individuals through square dance exercise can indirectly improve their cognitive function. The results of this study align with embodied cognition theory, which holds that human thoughts, perception and knowledge acquisition are achieved through body movements, sensations and experiences, i.e., that cognition is constructed through body experiences. Embodied cognition theory proposes that human cognition and physical exercise are closely connected [70]. Additionally, some studies have found that square dance exercise can improve the balance ability of elderly individuals. For example, a systematic review of older people revealed that square dance exercise improves balance ability, especially in terms of the ability to stand on one foot and gait control, thus improving cognitive function [71]. Another study on elderly women showed that square dance exercise significantly improved physical balance, cognitive function and mental health [72].

Neuroscience studies have shown that both balance ability and cognitive function are governed and regulated by the prefrontal cortex and hippocampus, and there is a close relationship and interaction between these two regions [73]. This further confirms that physical fitness and cognition are interrelated. Changes in one aspect will inevitably lead to changes in another, leading to changes in cognitive function. The results support our prediction that physical exercise improves cognitive function to some extent by improving physical fitness. Specifically, square dance exercise may lead to changes in the connectivity between the prefrontal cortex and the hippocampus by improving balance in older people. The prefrontal cortex plays a crucial role in the regulation of working memory, executive function and flexibility, while the hippocampus plays an important role in the formation of long-term memory and spatial cognition [74]. Therefore, square dance exercise may improve the connectivity between the prefrontal cortex and the hippocampus in elderly individuals, thereby promoting overall cognitive function. An interdisciplinary study also found that the balance ability of elderly individuals is closely related to the function of their nervous system, which can affect overall cognitive function [75]. It can be seen that square dance exercise not only improved the balance ability of elderly individuals but also uniquely improved overall cognitive development. Specifically, square dance exercise had a positive impact on the overall cognitive function of elderly individuals by improving their balance ability. This conclusion suggests new ideas and methods for the prevention and intervention of MCI in elderly individuals.

### The mediating effect of executive function on the relationship between square dance exercise and cognitive function

Executive function refers to a series of higher cognitive processes, including inhibitory, updating, and shifting processes, which can be achieved by brain regions such as the prefrontal cortex and the basal ganglia working in concert. According to the present results, executive function mediated the relationship between square dance exercise and cognitive function, indicating that the improvement in executive function by square dance exercise promoted overall cognitive function. The improvement in executive function may be another mechanism by which square dance exercise promotes overall cognitive function. In terms of physiological mechanisms, the improvement in executive function due to square dance exercise may be associated with several physiological mechanisms. First, square dance exercise may promote neural plasticity, increase the numbers and connections of neurons, improve the speed and excitability of neuron conduction, and ultimately improve executive function. Studies have found that square dance exercise significantly improves the executive function of elderly individuals, including working memory, cognitive flexibility and decision-making, and increases the hippocampal volume, thereby improving their spatial memory and spatial orientation and promoting overall cognitive function [76]. Second, square dance exercise promotes mental health by improving executive function. For example, square dance exercise can not only improve the executive function of elderly individuals but also reduce symptoms of anxiety and depression and improve overall mental health [77]. In addition, studies have shown that square dance exercise can significantly improve cerebral blood flow and brain function in elderly individuals, thus promoting executive function and overall cognitive function [78].

This study and the above studies all suggest that enhancing executive function can improve the overall cognitive function of elderly individuals with MCI. This further demonstrates that square dance exercise represents a beneficial nonpharmacological intervention that can directly improve the executive function of elderly individuals with MCI and indirectly prevent and treat MCI in elderly individuals.

### Chain mediating effect of physical fitness and executive function on the relationship between square dance exercise and cognitive function

The results showed that the effect of square dance exercise on the cognitive function of elderly individuals with MCI was achieved through the chain mediating effect of physical fitness and executive function. This chain mediating effect reveals the complex mechanism by which square dance exercise influences the cognitive function of elderly individuals and provides new ideas for further intervention research.

Specifically, square dance exercise improved overall cognitive function by promoting the physical fitness and executive function of elderly individuals. This influence may be realized through a variety of physiological and neurological pathways, such as the promotion of cerebral blood flow, increase in nerve cell production, and improvements in nerve conduction. Notably, the chain mediating effect revealed that the effect of square dance exercise on overall cognitive function is nonlinear and realized through multiple links and approaches. Therefore, practical intervention measures can be tailored to the physical fitness and cognitive function characteristics of each elderly person, and the most appropriate form and intensity of square dance exercise can be selected.

In conclusion, as a nonpharmacological intervention, square dance exercise can improve the cognitive function of elderly individuals, especially those with MCI, in a variety of ways and through a variety of mechanisms; this form of exercise has important preventive and therapeutic value. However, square dance exercise is only a supplementary intervention and needs to be combined with cognitive training, social interaction, psychological intervention and other comprehensive treatment programs. The combination of these methods with other types of physical exercise may maximize their effectiveness.

## Conclusions

Square dance exercise is effective in improving the level of mild cognitive impairment in older adults, mainly in terms of overall cognitive function. The improvement pathways were mainly mediated by balance and executive functions, respectively, as well as by a chain of mediation from balance ability to executive functions.

### Limitations and future research

Firstly, this study did not consider categorizing interventions for different subtypes of elderly people with MCI, and future interventions targeting subtypes of MCI should be conducted to increase intervention effectiveness and improve cognitive health in older adults.

Secondly, in this study, the sample size was small, and only one experimental group was designed for exercise intervention. Future studies may consider adding another exercise group (such as jogging, brisk walking or resistance training) to compare the intervention effects of square dancing with other form of exercise on the MCI population. Meanwhile, it is also necessary to consider exercise in conjunction with an integrated treatment program such as cognitive training, social interaction, and psychological interventions to maximize the intervention effect.

Finally, there are many other variables that affect cognitive function in older adults with MCI, such as social interactions and social relationships, which may also play a mediating role between exercise interventions and cognitive function. Future studies should explore the effects of these factors during exercise intervention.

## Data Availability

No datasets were generated or analysed during the current study.
